# Targeting Lipid Peroxidation for Cancer Treatment

**DOI:** 10.3390/molecules25215144

**Published:** 2020-11-05

**Authors:** Sofia M. Clemente, Oscar H. Martínez-Costa, Maria Monsalve, Alejandro K. Samhan-Arias

**Affiliations:** 1 Departamento de Química, Faculdade de Ciências e Tecnologia, Universidade Nova de Lisboa, 2829-516 Caparica, Portugal; smcclemente98@gmail.com; 2 Departamento de Bioquímica, Facultad de Medicina, Universidad Autónoma de Madrid (UAM), c/Arturo Duperier 4, 28029 Madrid, Spain; oscar.martinez@uam.es; 3 Instituto de Investigaciones Biomédicas ‘Alberto Sols’ (CSIC-UAM), c/Arturo Duperier 4, 28029 Madrid, Spain; mpmonsalve@iib.uam.es

**Keywords:** cancer, peroxides, LOX, COX, Fenton reaction, iron, ferroptosis, nanoparticles

## Abstract

Cancer is one of the highest prevalent diseases in humans. The chances of surviving cancer and its prognosis are very dependent on the affected tissue, body location, and stage at which the disease is diagnosed. Researchers and pharmaceutical companies worldwide are pursuing many attempts to look for compounds to treat this malignancy. Most of the current strategies to fight cancer implicate the use of compounds acting on DNA damage checkpoints, non-receptor tyrosine kinases activities, regulators of the hedgehog signaling pathways, and metabolic adaptations placed in cancer. In the last decade, the finding of a lipid peroxidation increase linked to 15-lipoxygenases isoform 1 (15-LOX-1) activity stimulation has been found in specific successful treatments against cancer. This discovery contrasts with the production of other lipid oxidation signatures generated by stimulation of other lipoxygenases such as 5-LOX and 12-LOX, and cyclooxygenase (COX-2) activities, which have been suggested as cancer biomarkers and which inhibitors present anti-tumoral and antiproliferative activities. These findings support the previously proposed role of lipid hydroperoxides and their metabolites as cancer cell mediators. Depletion or promotion of lipid peroxidation is generally related to a specific production source associated with a cancer stage or tissue in which cancer originates. This review highlights the potential therapeutical use of chemical derivatives to stimulate or block specific cellular routes to generate lipid hydroperoxides to treat this disease.

## 1. Introduction

Oxidative stress and inflammation are linked to cancer development [1,2]. Mutations in the DNA, phosphorylation of kinases, or inactivation of phosphatases can alter the cell growth, cellular control of the division, cell death, cell fate, and cell motility, which are altered in angiogenesis, inflammation, and fuel cancer progression [3,4,5,6]. A progressive increase of reactive oxygen species (ROS) marks the transition steps from a healthy tissue towards an invasive carcinoma [7]. This trend is owed to cancer cells’ metabolic aberrations to adapt strategies to escape from cell death. It occurs in the presence of compensatory upregulation of the genes coding antioxidant enzymes, preventing ROS induced cell death [2,5]. Therefore, a blockage of the antioxidant cellular defenses or pro-oxidant therapies’ stimulation is suggested as potential strategies to fight against cancer [8,9]. In general, lipids’ ability to participate in anti-inflammatory and/or pro-inflammatory signaling cascades is defined by: the length of the fatty acyl chain, the number of unsaturations, and the place where the oxidation account [10]. During lipid peroxidation, oxygen molecules are added to the unsaturated fatty acyl chain of non-polar lipids, increasing their water solubility and diffusion towards the membrane surface. Cyclooxygenases or lipoxygenases accessibility for their substrates is boosted, the generation of lipid metabolites linked to inflammation is prompted, and the interaction of specific proteins and receptors recognizing lipid oxidation products is promoted [11,12,13,14,15]. All of these actions are part of the lipid-dependent inflammatory cascade. Moreover, these arrangements prevent the accumulation of lipid peroxides into the membrane, which might functionally damage membrane components such as proteins by induction of covalent modifications, which might compromise membrane permeability [16,17]. In general, free fatty acids can be released from phospholipids by phospholipases, and this pathway is essential in inflammation, since it counterparts the activities of lipoxygenases and cyclooxygenases [18]. Polyunsaturated fatty acids (PUFA) and their related phospholipids are very well-known signaling molecules with pro-inflammatory and anti-inflammatory functions, but also sensitive substrates for peroxidation [19,20,21,22,23,24,25,26,27,28]. PUFA can promote cell life or cell death through complex signaling cascades related to the fatty acid structure and their oxidation products [29,30]. In cancer, the connection of some of these pathways with inflammation is unveiled by identifying a group of lipid oxidation products, known as lipid pro-resolving mediators, which can resolve the inflammation [31,32,33]. Their discovery opens the opportunity to identify new potential drugs in cancer therapy [32]. Some lipid oxidation products have also gained attention since they are suggested as biomarkers for cancer development and recurrence [34,35]. In general, lipids’ ability to participate in anti-inflammatory or pro-inflammatory signaling cascades depends on the lipid’s nature and its degree of oxidation. In this review, we dissect the lipid hydroperoxides and metabolites sources found in cancer research to better define proper actions to treat the malignancy and highlight those stimulated pathways found in tumors from those triggered in strategies to kill cancer cells.

## 2. Lipid Peroxidation: Non-Enzymatic Reactions vs. Enzymatic Reactions

Cellular lipid peroxidation can occur through different reactions, but they can be categorized into enzyme and non-enzyme dependent reactions. The primary substrates in lipid peroxidation reactions are polyunsaturated lipids since carbon-carbon double bonds are susceptible to reactive oxygen species, such as the hydroxyl radical (HO^•^), which is a key radical that participates in peroxidation reactions.

### 2.1. Non-Enzyme-Dependent Lipid Peroxidation

In the non-enzymatic reactions, the Fenton and Haber–Weiss reactions producing HO^•^ are dependent on transition metals (i.e., iron [36,37]), for the initiation of the radical chain reactions required for lipid peroxidation. In addition to this radical formation, some authors have suggested that for initiation of lipid peroxidation, the formation of a complex between iron and the lipids is required [38]. In general, it is accepted that the initiation reaction starts when a hydrogen atom is abstracted from lipid, forming an alkyl radical [39]. HO^•^ is preferred over other radicals to performed this abstraction [38,39,40,41,42,43]. Once the alkyl radical is formed, the chain-carrying a carbon radical reacts with oxygen, leading to an alkyl peroxyl radical formation. This radical can abstract hydrogen from an organic substrate, which can be another lipid, to form a hydroperoxide plus an organic radical or be added to alkenes, such as those present in the fatty acyl chains of PUFA present in phospholipids, which provide isolated double bonds [44]. This last reaction leads to the formation of a lipid hydroperoxide with a conjugated double bond. By reaction with metals, lipid radical reactions leading to lipid peroxidation can be reinitiated as part of the propagating radical reactions [45,46]. This process occurs when the hydroperoxides react with an oxidized metal forming an alkoxyl radical. In case the reaction involves a reduced metal, e.g., Fe^2+^, an alkyl peroxyl radical is generated, which also contributes to the propagation of the reaction. PUFA are lipid molecules priming the Fenton’s reaction, as previously indicated. Arachidonic acid (AA) and the phospholipids containing this fatty acid are essential molecules since they are precursors of pro- and anti-inflammatory mediators, sometimes enriched at cellular locations identified as signaling platforms, such as the plasma membrane lipid rafts [47,48]. Lipid oxidation at this location during the inflammation process is relevant since lipid rafts are platforms required for cell activation in the immune system [49].

### 2.2. Radiation Inducing Lipid Peroxidation

HO^•^ can also be generated by ionizing radiation [38], which is generally used and applied in patients to treat cancer. As previously indicated, HO^•^ is a very reactive radical, leading to the generation of lipid hydroperoxides and oxidizing other biomolecules, including the DNA. Radiation exposition leads to peroxides generation in membranes enriched with PUFA. Indeed, this fact should not be discarded as a relevant factor for cancer therapy’s success in these patients [50,51,52,53]. Noteworthy, more efforts are required to shed light on the role that ionizing radiation generating lipid peroxides have in cancer cell death vs. other targets. These investigations could help in the characterization of pharmacological drugs prompting the cancer cell sensitivity to lipid hydroperoxides generated by ionizing radiation (i.e., glutathione peroxidase 4 (GPX4) inhibitors) and, therefore, better define or reduce the patient’s exposure to ionizing radiation that can also damage non-tumoral tissues.

### 2.3. Enzyme-Dependent Lipid Peroxidation 

The enzyme-dependent reactions are executed by peroxidases, which have been elegantly classified by Vlasova [54]. Based on this classification, lipids can be oxidized by proteins that possess a true peroxidase activity, such as lipoxygenases (LOX), cyclooxygenases (COX), and cytochrome P450, or by proteins that do not have a peroxidase activity but acquire a pseudo-peroxidase activity under certain conditions, i.e., cytochrome *c* [55,56,57,58,59] upon binding to cardiolipin or other hemeproteins in defined conditions [60,61,62,63]. The coordinated iron or iron associated with the heme group is key in these enzymes’ catalytic center. Compound I, compound II, and sometimes compound III are generally typical and associated with different iron valences [64]. The function of real peroxidases might depend upon the existence of binding pockets where substrates can settle and interact with the enzyme catalytic center, and upon electrons donated by organic molecules, which might be protein amino acids acting as electron donors [54]. In heme-dependent pseudo-peroxidases, substrates accessibility to the enzyme coordinating sphere depends upon the catalytic center flexibility to swift from a metal hexa- to penta-coordination, a feature that can be influenced by the redox state or the interaction with a ligand, i.e., cytochrome c upon cardiolipin binding to the protein [55]. The main enzymes using AA to generate lipid hydroperoxides and derived metabolites as signaling molecules in cancer are cyclooxygenases (COX), lipoxygenases (LOX), and P450 families [29,30,47,65]. COX-1 is constitutively expressed in many tissues and cell types, whereas COX-2 is an inducible cyclooxygenase isoform which activation has been reported in tumoral tissues [66,67,68,69,70,71]. Some studies have also pointed out that other peroxidases (like myeloperoxidase and eosinophil peroxidase) released from infiltrating neutrophils and eosinophils in the tumor microenvironment [72,73,74,75] or from infiltrated macrophages can also generate lipid hydroperoxide [76]. Although myeloperoxidases are potential sources of lipid peroxides and some myeloperoxidases polymorphisms, have been correlated with a higher risk of suffering pulmonary, ovarian, and gastric cancer [77,78,79], there is no correlation between their activity and the lipid peroxides derived from it with disease development.

**Figure 1:**
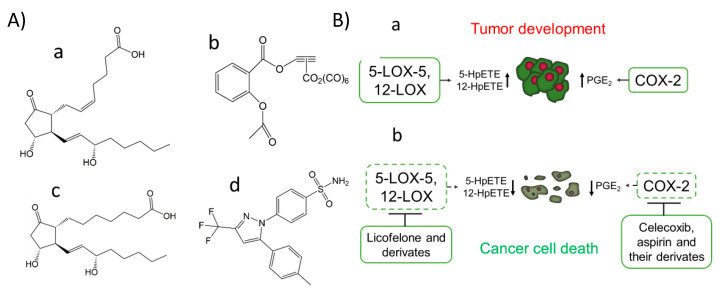
COX-2 inhibitors, PGE_1,_ and PGE_2_ chemical structure (**A**). The prostaglandin E_2_ (PGE_2_) (pro-cancer) (**a**), the aspirin derivate named [2-acetoxy-(2-propynyl)benzoate]hexacarbonyldicobalt (Co-ASS) (**b**), the prostaglandin E_1_ (PGE_1_) (anticancer) (**c**) and the COX-2 selective inhibitor named celecoxib, (*p*-(5-*p*-Tolyl-3-(trifluoromethyl) pyrazol-1-yl)benzenesulfonamide) (**d**). Expression of COX-2, 5-LOX, and 12-LOX in cancer and the effect of inhibitors against these targets (**B**). Activation of 5-LOX-5, 12-LOX, and COX-2 has been reported in the development and progress of tumors from different tissues associated with the production of specific lipid peroxides and metabolites, such as PGE_2_ (**a**). Some inhibitors of these LOX isoforms and COX-2 have emerged as potential therapeutical agents for cancer treatment, modulating the production of previously commented metabolites and by induction of cancer cell death (**b**).

In contrast, anti-inflammatory drugs have been linked to a decreased risk of cancer development and decreased tumor growth rate [28]. Notably, in this context, overexpression of enzymes generating eicosanoids in breast, lung, and pancreas cancer has been reported [28]. In particular, prostaglandins (PGs) can stimulate mitogenesis by directly affecting fibroblasts, osteoblasts, and mammary cells. The production of the proinflammatory PG named prostaglandin E_2_ (PGE_2_) (Figure 1A) through COX-2 activity can be found in mutagenesis, angiogenesis, and cell migration processes associated with cancer (Table 1). An activation mechanism for COX-2 has been proposed using human colorectal HT-29 and the human prostate carcinoma DU145 cell lines [80]. A correlation between the production of PGE_2_ with the resistance of cancer cells to apoptosis has been found through activation of the P2Y_2_/Src/p38 signaling pathway, which lead to AA release from the membranes by overexpression of some PLA_2_ isoforms, and the overexpression of COX-2 with the subsequent PGE2 production [80].

To define the role of lipid peroxidation in signaling, the type of lipid hydroperoxide generated should be finely characterized. In the metal-mediated lipid peroxidation based on the Fenton and Haber–Weiss reactions, random lipid peroxides are generated and differentiated from those formed by specialized enzymes that produce specific lipid signatures that can be used as fingerprints of enzymatic activities [58,81,82]. Many efforts are being made to find specific inhibitors that can provide a specific modulation of lipid hydroperoxide production that could act as mediators in signaling cascades [58,83]. For example, aspirin, which has beneficial effects in some cancer types, has been proposed to play that role [82,84,85]. Its implication in cancer has been associated with COX inhibition via acetylation of the active site, where AA binds. This accounts for COX-1 isozyme inhibition, while in COX-2, aspirin binding produces a structural rearrangement shifting the cyclooxygenase towards the lipoxygenase activity [86,87]. Therefore, AA oxygenation and cyclization to form a 15*R*-Prostaglandin endoperoxide is promoted, which favors the production of Prostaglandin D_2_ (PGD_2_) (with has a suggested function in inflammation resolution) instead of PGE_2_ [88]. Recent studies have also shown the potential therapeutically effect of aspirin organometallic derivatives as anticancer agents targeting COX-2 [89], such as the 4-[5-(4-Chlorophenyl)-3-(trifluoromethyl)-1*H*-pyrazol-1-yl]-benzenesulfonamide (SC-236) and [2-acetoxy-(2-propynyl)benzoate]hexacarbonyldicobalt (Co-ASS) (Figure 1A), which open a promising field in the search for inhibitors derived from aspirin [90,91,92].

**Table 1:** Correlation between COX-2 level and other biomarkers found in tumoral tissues

Tissue Location and Type of Cancer	Correlation with Other Biomarkers
Colon cancer [66], primary tumors and metastatic lymph nodes resections for colorectal adenocarcinoma [93], stage II and III colorectal cancer patients [94]	High levels of COX-2 correlates with high levels of MMP-2 and VEGF expression and shorter survival time [93,94].
Cervical cancer [67]	Multivariate analysis of COX-2 levels in tumor/stromal compartments. The proportion of CD3^+^, CD4^+^, and CD25^+^ cells was lower in tumors with high tumor/stroma ratios, but in these tumors, mast cells were increased [67].
Ovarian cancer [95,96,97]	No correlation between COX-2 expression and EGFR, and HER-2/neu status [96].
Human breast cancer cell lines and tumors [26,98,99,100]	Elevated COX-2 expression associated with a large tumor size, a high histological grade, a negative hormone receptor status, a high proliferation rate, high p53 expression, and the presence of HER-2 oncogene amplification along with axillary node metastases and a ductal type of histology [98]. COX-2 inhibition may potentially prevent the development of ER-positive and ER-negative breast cancers [98]. Expression of PGE_2_ and IL-8 [101]. COX-2 over-expression induces an oncogenic microRNA (miR655) in human breast cancer cells by activation of EP4 [102].
Ductal carcinoma in situ (DCIS) [103,104,105]	COX-2 expression stabilizes survivin, an inhibitor of apoptosis (IAP) [103]. CacyBP expression was significantly negatively associated with the COX expression [104].
Non-small cell lung cancer [68,69]	Correlation between HER-2, EGFR, and COX-2 expression in patients of non-small cell lung cancer at different degrees [69]
Laryngeal cancer [71]	Cox-2 overexpression was significantly associated with radioresistant tumors [71].
Papillary thyroid cancer [106]	The expression of COX-2 is increased with age in papillary thyroid cancer [106]. Immunohistochemically, expression of COX-2 and VEGF-C correlated strongly, and both were induced by the tumor promoter phorbol 12-myristate 13-acetate [107].
Endometrial hyperplasia and carcinoma [108,109,110]	No correlation between COX-2 expression with estrogen (ER) or progesterone receptor (PR), p53, and neu [110]. Correlation between COX-2 (59%) and aromatase (65%) expression but not estrogen and progesterone receptor [111].
Invasive gallbladder cancer [112]	COX-2, c-Met, β-catenin, c-erbB2 and EGFR were over-expressed in 80%, 74%, 71%, 62%, and 11% of invasive gallbladder cancers, respectively [112].
Prostate cancer Metastatic primary prostate carcinoma compared to non-metastatic cancers [113,114,115,116]	COX-2 and Ki-67 antigen co-expression in 42.9% and 67% of the prostate cancer patients [113]. Patients with PSA > 7 ng/mL and high COX-2 expression had the highest probability of recurrence [114]. The expressions of COX-2 and E-cadherin are very firmly and inversely correlated as prognostic indicators. [115]. High expression of COX-2, TGF-beta, and Ki67 in metastatic primary prostate carcinoma was associated with death from prostate carcinoma [116].
Gastric cancer [117,118]	A positive correlation between COX-2 and K-ras expression with the depth of invasion and lymph node metastasis in gastric cancer [117]. Epithelial MMP-2 expression in gastric cancer is associated with aggressive forms, COX-2 expression, and poor survival [118].
Cervical cancer [119]	DNA hypermethylation of the COX-2 gene may be a potential prognostic marker in the early stages of cervical cancer [119].
Pancreatic cancer [120,121] Anaplastic pancreatic cancer [122]	Tumor COX-2 expression portends a poor prognosis for patients with resected adenocarcinoma of the pancreas, particularly in tumors > or = 3 cm [121]. Expression of L1CAM, COX-2, and EGFR in the majority of undifferentiated pancreatic carcinomas [122].

### 2.4. Lipid Peroxidation Derived Products and Biological Targets

Lipid hydroperoxides generated via enzyme or non-enzyme-dependent reactions can be further oxidized to form highly reactive species and lipid autoxidation products. Acrolein, malonaldehyde, and 4-hydroxynonenal can covalently modify proteins leading to functional and structural changes in proteins [123,124,125]. Lipid autoxidation products mainly react with primary amines and lysines, histidine, and cysteine residues from proteins to induce covalent crosslinking and prompt protein aggregation [125]. The amino acid residues mentioned above are also the primary targets for several protein post-transductional modifications, such as acylation, acetylation, phosphorylation, methylation, glycation, and S-nitrosylation, among other modifications [126,127]. Therefore, it can be presumed that the reaction of essential amino acid residues with lipid autoxidation products will also induce changes in the signaling pathways in which these proteins are involved. The generation of lipid autoxidation products has been reported in cancer development, angiogenesis, and invasiveness [128,129,130,131,132]. Some autoxidation products, such as 4-hydroxynonenal, have been implicated in DNA modifications that generate cancer-linked mutations [1].

### 2.5. Antioxidants against Lipid Radical Reactions and Peroxidases

Antioxidants play a central role to counteract lipid peroxidation. In the non-enzyme-dependent reactions, the radical chain reactions can terminate when antioxidants react with the alkyl peroxyl or the alkoxyl radicals. Tocopherol is the main membrane antioxidant in charge of reacting with these radicals and one of the primary membrane antioxidants against reactions generating lipid peroxides, in general [133]. Consequently, a lipid hydroperoxide or the alcohol and the radical antioxidant are products of the reaction between the lipid radical with the antioxidant. In the particular case of the tocopheroxyl radical, it can be reduced back to tocopherol by its reaction with other antioxidants, such as ascorbate and ubiquinol [134], or by enzymes in charge of reducing the antioxidant radical [135,136]. Indirectly or directly, the enzymatic activities that reduce the radicals derived from antioxidants are essential to keep optimal alpha-tocopherol levels in the membrane [135,137,138]. Other types of enzymes, such as glutathione peroxidases, can reduce lipid hydroperoxides to alcohols at the expense of glutathione (GSH). GPX4 is a pharmacological target in cancer [139,140,141], and its inhibition has been found to induce cancer cell death by the accumulation of lipid hydroperoxides [140].

## 3. Lipid Hydroperoxides Generated by Stimulated Lipoxygenases (LOXs), Cyclooxygenases (COXs), and the Role of Their Metabolites in Cancer

### 3.1. COXs

The main AA oxidation products of COXs activity are PGs. Prostaglandin H_2_ (PGH_2_) is a PG generated by both COX-1 and -2 using AA as a substrate, which acts as a precursor for the generation of other PGs such as the thromboxane A_2_ (TxA_2_) and the PGs named PGI_2_, PGD_2_, PGE_2_, and PGF_2_α [142]. PGs are important in the early inflammatory response, and their production is increased in the inflamed tissue mainly by stimulation of COX-2 activity [143]. In addition to COXs, other pathways have been implicated in the generation of PGs in cancer, such as those dependent upon prostaglandin synthetases that might be functionally coupled to COX-2 activity and, in some cases, might also be dependent upon glutathione [23,144,145]. In general, raised levels of COX-2 and 12-lipoxygenase (12-LOX) in patients who developed metastatic disease or local recurrence and/or died have been found in other studies and, therefore, these enzymes have been proposed as biomarkers in cancer [146,147]. COX-2 in combination with other biomarkers has also been used in cancer prognosis (Table 1). In addition, a strong correlation between the production of PGE_2_ and cancer development has been observed [34,35].

For the generation of PGE_2_ by COXs, a peroxidation and cyclooxygenation reaction of the substrate is required. On the distal side from the heme moiety, an array of amino acids provides the site for hydroperoxides to bind and assists the heme-dependent reduction of hydroperoxides to alcohols [148]. Therefore, the formation of the PG involved in cancer named PGE_2_ requires several steps for its production [23]. First, the formation of unstable endoperoxide intermediate named prostaglandin G_2_ (PGG_2_) through COX activity using AA as a substrate. This PG can be further metabolized to a second unstable endoperoxide intermediate named PGH_2_, which can be enzymatically broken down to generate the PGE_2_ [23,24,25,26,27,28]. 

PGE_2_ acts binding to any of the specific G-coupled receptors belonging to four subclasses, EP1-EP_4_, which have specific signal transduction activities, tissue localization, and regulation [149]. The activation of each of the different receptors and downstream signaling cascades makes PGE_2_ activity highly dependent on the specific docking receptor [150]. The role of each EP receptor in malignant behavior is complex, but their signaling cascades have been found linked to different stages of tumoral and immune processes [151].

Moreover, downstream metabolites derived from PGE_2_ are compounds, such as 15-keto-PGE_2_, generated by 15-hydroxyprostaglandin dehydrogenase [152,153]. This compound ligates to the transcription factor peroxisome proliferator-activator receptor gamma (PPARγ), which regulation has been found to regulate genes with anti-proliferative and anti-inflammatory effects and play a protective role against tumor development [154,155]. Some PPARγ agonists, including 15-keto-PGE_2_ and thiazolidinediones, have been proposed as potential pharmacological therapeutical drugs in cancer [156,157,158,159]. However, it is still unclear whether PPARs act as bona fide tumor suppressor or as an oncogene, and more studies are needed to understand their role in cancer for the development of efficient and safe chemotherapeutic agents targeting these molecules [154]. Other AA derived metabolites, such as 15-deoxy-delta-12,14-prostaglandin-J_2_ (15d-PGJ_2_), are also known as PPARγ agonists. Besides, PGD_2_, PGJ_2_, Δ^12^-PGJ_2_ are also known to induce the apoptosis of tumoral cells [160,161].

### 3.2. The Use of COX Inhibitors to Induce Cancer Cell Death

The possibility of product formation modulation by altering the substrate availability has traditionally been the primary COX activity regulation approach. Some substrates of COX-2 activity other than AA, such as γ-linolenic acid and dihomo-γ-linolenic acid, have been demonstrated to pose anti-cancer effects and have been proposed as promising dietary supplements for cancer prevention since they increase the formation of PGE_1_ (Figure 1A) and related metabolites, which have anti-inflammatory properties [19,20,21,22]. Additionally, pharmacological drugs targeting the COX/LOX have been identified. Combined inhibition of 5-LOX/COX-2 has been used to treat some types of cancer [162,163,164,165,166]. COX-2 inhibitors such as celecoxib (Figure 1A,B), aspirin, diosgenin, and ibuprofen have been proposed to have anti-tumor activities, being useful in preventing and treating several cancer types [99,167,168,169,170]. Noteworthy, in some instances, a lack of response to COX-2 inhibitors, such as aspirin in some types of cancer at low concentrations, has been reported despite the potential noted benefit among individual patients population with stage I tumors [171,172]. However, some reports have evidenced a lack of effectiveness of this compound due to the upregulation of 15-LOX-1 that might be induced to compensate the lack of some lipid oxidation products [173].

### 3.3. LOXs

This group of enzymes is named based on the place of the carbon chain where the oxidation occurs. Six functional LOX genes (ALOX5, ALOX12, ALOX15, ALOX15B, ALOX12B, and ALOXE3) have been identified in humans [174]. Historically, mammalian LOXs are classified based on the position where AA oxygenation occurs [175]. Classification of genes, expression, and tissue location can be found in Table 2.

The 12-LOX utilizes AA to synthesize 12(*S*)-hydroperoxyeicosatetraenoic acid (12(*S*)-HpETE), which is converted to the end-product named 12(*S*)-hydroxyeicosatetraenoic acid (12(*S*)-HETE) implicated in the promotion of tumorigenesis, proliferation, and metastasis by stimulation of the vascular endothelial growth factor (VEGF), and some integrins expression and controlling the cell cycle [177,178,179,180]. Besides 12(*S*)-HETE, metabolites generated from 5-LOX such as 5-hydroxyeicosatetraenoic acid (5(*S*)-HETE), which precursor is 5(*S*)-HpETE, have been implicated in the stimulation of prostate cancer cell growth [181]. Upregulation of 5-LOX and 12-LOX activities in cancer contrast with the decrease in 15-lipoxygenase (15-LOX) isoform 1 (15-LOX-1) function, as well as in the metabolites generated from linoleic acid oxidation, such as 13–hydroperoxyoctadecadienoic acid (13(*S*)-HpODE) [178] and 13-*S*-hydroxyoctadecadienoic acid (13-*S*-HODE), which are reported in human colorectal and esophageal cancers [173]. These effects might be related to a downregulation of the 15-LOX-1 gene expression or its inactivation, as demonstrated for colon cancer [182,183,184,185], where the transcription factor GATA-6 is responsible for this effect [183]. Other derived hydroperoxides from 15-LOX-1, such as hydroperoxyoctadecatrienoic acid (13-HpOTrE), 13-HpODE, and 15-hydroperoxyeicosatetraenoic acid (15-HpETE) have been reported as inhibitors of breast, colon, prostate, lung, and leukemia cancer growth in conventional cell studies in vitro [186], supporting that stimulation of this 15-LOX-1 could be potentially used for therapeutical purposes. Among these compounds, 13-HpOTrE is reported to be the most active hydroperoxide regarding cytotoxicity and apoptosis induction in cell culture experiments [184,186]. Notably, other than in the two-dimensional (2D) culture experiments, 13-HpOTrE treatments for as long as a week did not show significant effects on cell viability in 3D cell culture experiments of tumor cells but resulted in decreased IL-6 release [186]. These results support the specific role of some lipid hydroperoxides as pro-survival or cell death signals in cancer that could be modulated by a specific tissue microenvironment and linked to the specific LOX isoforms. 

Moreover, 15-LOX-1 activity is elevated when ferroptosis is induced in cancer cells [139]. Some of the main phospholipids acting as substrates for 15-LOX-1 in the context of ferroptosis are phosphatidylethanolamines (PE) containing PUFA in their fatty acyl chain, which generates lipid hydroperoxide and other PE oxidation products as ferroptosis signals [187]. This target specificity is associated with phosphatidylethanolamine-binding protein 1 (PEBP1) activity that acts as a scaffold protein inhibitor of protein kinase cascades that can form a complex with some 15-LOX isoforms [188]. 

### 3.4. The Use of LOX Inhibitors to Induce Cancer Cell Death

The nonsteroidal anti-inflammatory drugs acting as inhibitors of COX/LOX named tepoxalin [189], and licofelone [190] have been successfully used to halt the progression of gastric cancer and colon cancer cells, respectively, in tumor xenograft mice. These results suggest that inhibition of certain LOXs that have been found stimulated in cancer might be used to block cancer development. The most potent inhibitor of 12-LOX, named Zyflamend, presents cancer antiproliferative activity in human prostate cancer PC3 cells [191]. Peptides against 12-LOX, such as the one formed by tyrosine, tryptophan, cysteine and serine residues (YWCS), have also been developed and suggested for breast cancer treatment, and specific 12-LOX inhibitors, such as baicalein, due to their tumor-suppressive and anti-angiogenesis effects [192,193]. Other therapeutical approaches have demonstrated the efficacy of 12-LOX inhibition in vitro and in vivo experiments using human prostate cancer cells [194,195]. To summarize, it seems that the combinatorial effect of these enzyme inhibitors with those of COX-2 can prevent the generation of tumorigenic signaling molecules derived from 5-LOX and 12-LOX, whose synthesis is induced in some types of cancers with pathological consequences (Figure 1B).

## 4. Stimulation of Peroxidases to Induce Cancer Cell Death: The Case of 15-LOX-1 Activity in Ferroptosis

In cancer, lipid peroxidation became interesting for researchers since its involvement, and implication in cancer cell ferroptosis was discovered [196]. Many well-written reviews are describing this type of cell death [82,197,198]. Essentially this process can be summarized as a cell-regulated iron-dependent form of non-apoptotic cell death derived from lipid hydroperoxide accumulation [199], more predominantly phosphatidylethanolamine hydroperoxides [187]. In ferroptosis, iron chelators, lipophilic antioxidants, lipid peroxidation inhibitors, and depletion of polyunsaturated fatty acids (PUFAs) can block this cell death process [200]. Inhibition of GPX4 activates ferroptosis, and since this enzyme function is dependent upon GSH that acts as a substrate for this activity, those processes that limit GSH biosynthesis are essential and also to trigger this type of cell death [200,201]. For example, inhibition of the cystine/glutamate antiporter system xc- regulates the transsulfuration pathway, in charge of cysteine biosynthesis, and limits the synthesis of glutathione [200,201]. In correlation with this point, glutamate and glutamine deficiency also regulate ferroptosis [202]. Besides GPX4 activity depletion, suppression of some non-steroidogenic metabolites of the mevalonate pathway enhances some ferroptosis inducers’ sensitivity, such as the ferroptosis activator named FIN56, which acts independently of GPX4 degradation [203]. From this pathway, ubiquinone has emerged as an essential molecule that could modulate ferroptosis [203], which correlates with the early described function as one of the major cellular antioxidants against lipid peroxidation [204,205,206,207]. Ferroptosis is also dependent upon stimulation of several other enzymatic processes, such as the biosynthesis of PUFA-containing phospholipids that are the primary substrate for selective oxygenation by lipoxygenases, the acyl-CoA synthetase long-chain family member 4 (ACSL4), in charge of free fatty acids conversion into fatty CoA esters, and the lysophosphatidylcholine acyltransferase 3 (LPCAT3), which is involved in phospholipid biosynthesis [200]. Iron can also mediate the activation of ferroptosis, and processes and iron import, export, storage, and iron turnover impact the cell sensitivity to ferroptosis [200]. These processed should be summed to the fact that iron is an enzyme effector for non-heme dependent enzymes such as LOXs in charge of the generation of lipid peroxidation products [187,208,209]. Although iron chelators and genetic inhibition of cellular iron uptake can block ferroptosis [210,211], the increase in H_2_O_2_ production dependent upon iron can not only be attributed to Fenton’s chemistry [199]. All 15-LOX isoforms, but 15-LOX-1, significantly [187,209], play a crucial role in generating lipid hydroperoxides associated with ferroptosis. Iron has also been implicated in this process since iron chelators can rescue cells from experimental induction of ferroptosis [210,211]. Therefore, there is some controversy on the relative role of enzymatic and non-enzymatic metal-dependent reactions in the generation of lipid hydroperoxides. The combined activity of 15-LOX and iron-binding proteins in ferroptosis are likely to be required to generate phospholipid autoxidation products that participate in the initiation-propagation of the lipid radical chains implicated in the modification of cellular regulatory proteins driving ferroptosis [82,212,213]. Moreover, the activity of the phosphorylase kinase G2 (PHKG2), a key enzyme in the control of cellular iron levels, is necessary to induce ferroptosis in the erastin-induced model, where LOX induced formation of lipid hydroperoxides has been demonstrated as one of the main inducer mechanism of ferroptosis [209].

## 5. Blockage of Antioxidant Enzymes to Increase Lipid Peroxidation in Tumoral Cells

### 5.1. GPX4

**Figure 2:**
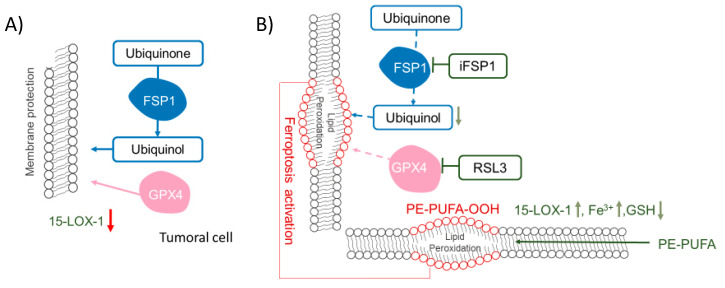
Glutathione peroxidase 4 (GPX4) and ferroptosis suppressor protein 1 (FSP1) function in cancer cells. Role of ubiquinone reduction through FSP1 and GPX4 in the protection of the membrane against lipid peroxidation in cancer cells (**A**). In cancer cells, the antioxidant enzymes GPX4 and FSP1, which act on reducing lipid hydroperoxides, would protect them against cell death. Lipid peroxidation and stimulation and ferroptosis induction by the FSP1 and GPX4 inhibitors’ such as iFSP1 and RSL3, respectively (**B**). By the blockage of these enzymes and stimulation of LOXs, such as 15-LOX-1, lipid peroxidation is prompted. Moreover, substrates of these enzymes and mediators such as GSH and iron could stimulate ferroptosis in cancer cells.

Ferroptosis can be triggered by inhibition of GPX4 (Figure 2), a peroxidase in charge of reducing lipid peroxides at the expense of GSH [140]. Therefore, ferroptosis in cancer can be triggered by inhibition of this enzyme [140], and some efforts have been made to find specific inhibitors; (1*S*,3*R*)-RSL-3 (RSL3) was the first described irreversible inhibitor of GPX4 [140,199,209]. Other inhibitors, such as ML210 and ML162, are mesenchymal state-targeting compounds inhibiting GPX4 [214]. Due to GPX4 dependence upon GSH, compounds downregulating cellular GSH levels (such as erastin and sorafenib [199], as well as inhibitors of the xc-cystine/glutamate exchanger system that limits the novo synthesis of GSH [215]), can also promote ferroptosis. GSH depletion also leads to ferroptosis activation in cancer cells and the accumulation of lipid hydroperoxides [216]. Moreover, the ferroptosis suppressor protein 1 (FSP1), an enzyme in charge of ubiquinone reduction, has emerged as a potential pharmacological target to inhibit cellular ferroptosis resistance in the GPX4 deficiency model (Figure 2) [217].

### 5.2. Quinone Reductases (QRs)

Several recent studies have recently evidenced the role of QRs in cancer cells using a GPX4 deficiency model and have highlighted the potential of ubiquinol and, therefore, QRs as novel targets for cancer treatment (Figure 2). Their role in cancer seems to be related to their capacity to reduce ubiquinone. Reduced ubiquinone is necessary to recycle oxidized alpha-tocopherol and protect membranes from lipid hydroperoxides [204]. Studies using inhibitors of the mevalonate pathway, blocking the ubiquinone synthesis, have evidenced the ubiquinone role in this type of cell death [203]. Some QRs, including the NAD(P)H Quinone Dehydrogenase 1 (NQO1), have been shown to work with a *K*_m_ value for quinones in the same range reported for FSP1 [217]. Although it is known that NQO1 cannot compete with FSP1 in the reaction with hydrophilic peroxides, the situation is reversed when the substrate is lipid hydroperoxides. Other QRs, with similar or higher *K*_m_ values for quinones, are also putative targets for cancer treatment combined with GPX4 inhibitors. We proposed a list of described mammalian QRs with reported *K*_m_ values, the name and structure of the specific inhibitors available for these enzymes, and the IC_50_ value reported for cancer cells as potential targets to inhibit to promote ferroptosis in conjunction with GPX4 inhibitors (Table 3).

## 6. The Future of Nanoparticles (NP) for Cancer Targeting and Tissue Specificity

In the last decade, NPs have emerged as tools for delivering compounds for cancer treatment. Up to date, attempts to generate NPs loaded with lipid hydroperoxides have been hampered due to the high reactivity and instability of lipid hydroperoxides, hindering the proper delivery of intact peroxides into cancer cells. Noteworthy, and based on the evidence accumulated using the pharmacological targets reviewed here, the use of NPs to facilitate the distribution of drugs modulating lipid hydroperoxide generation in cancer patients seems to be of potential pharmacological interest. NPs can incorporate molecules on their surface that preferentially target cancer cells (i.e., folate [232] and 5-(4-hydroxyphenyl)-10,15,20-triphenylporphyrin [233]). The successful delivery of anticancer drugs can be achieved through this strategy [234,235,236,237].

Some recent advances have been made regarding the synthesis of NPs loaded with metals, mainly with iron, which can facilitate lipid peroxidation in cancer cells [234,238,239]. As previously indicated, iron overload produces an increase in random peroxidation products that is entirely unspecific and could lead to harmful oxidative stress in non-cancer cells. Ferroptosis induction has been indirectly associated with the use of pharmacological inhibitors of this type of cell death. However, in some cases, the mechanism of action that induces ferroptosis is unknown and does not seem mediated by inhibition of antioxidant enzymes [237]. Due to iron’s high reactivity, GSH oxidation induced by the metal should be considered a therapeutical approach and could be the main triggering factor leading to ferroptosis activation. Some reports have been published describing ferroptosis induction thought combination therapy where iron NPs are conjugated with ferroptosis inducers, such as the xc-cystine-glutamate exchanger’s inhibitor Sorafenib, that induces glutathione depletion [240], as well as with drugs promoting lysosomes disruption [241,242].

Regarding COX inhibitors, the use of nebulized colloidal poly d,l-lactide-co-glycolide (PLGA) nanoparticles co-encapsulating a COX-2 inhibitor (celecoxib) and a herbal compound (naringenin) has shown promising results for lung cancer treatment in in vitro studies [243]. A similar strategy for the treatment of tumoral glial cells has been reported [244]. As pertaining to LOX inhibitors, we did not find specific reports on their use in combination with NPs to treat cancer. Although some authors reported the photodynamic therapeutic effect of indocyanine green entrapped nanoparticles in skin cancer and the inhibition of COX-2 and 5-LOX, the inhibition mechanism involved remains unknown. More efforts should be made to evaluate the potential applications of NPs to deliver LOX inhibitors for cancer treatment.

## Figures and Tables

**Table 2:** Human lipoxygenases (LOXs) genes classification and expression in cells and tissues.

ALOX Gene	Name	Cell and Tissue Location
ALOX5 PubMed Gene ID: 240	arachidonate 5-lipoxygenase or 5-lipoxygenase (5-LOX)	Monocytes, macrophages, B lymphocytes cells [175] and appendix, bone marrow, gall bladder, lung, lymph node, spleen, and urinary bladder [176].
ALOX12 PubMed Gene ID: 239	arachidonate 12-lipoxygenase, 12S type or 12-lipoxygenase (12-LOX)	Esophagus and skin [176].
ALOX15 PubMed Gene ID: 246	arachidonate 15-lipoxygenase or platelet type platelet lipoxygenase or 15-lipoxygenase-1 (15-LOX-1)	Reticulocytes, eosinophils [175] and lung, small intestine, testis urinary bladder [176].
ALOX15B PubMed Gene ID: 247	arachidonate 15-lipoxygenase type B or 15-lipoxygenase-2 (15-LOX-2)	Human skin [175] and prostate, lung, and esophagus [176].
ALOX12B PubMed Gene ID: 242	arachidonate 12-lipoxygenase, 12R type or 12R-lipoxygenase (12R-LOX)	Skin and esophagus [176].
ALOXE3 PubMed Gene ID: 59344	arachidonate lipoxygenase 3, lipoxygenase, epidermis type (eLOX3)	Skin, tongue, prostate, tonsils [175,176].

**Table 3:** Summary of enzymes with Quinone Reductases (QR) activity, reported kinetic properties, and implication in cancer.

Name	*K*_m_ (CoQ) (µM)	Specific Inhibitor with Anti-Cancer Properties	IC_50_ (µM) and Cancer Cell Line	Structure
FSP1 (AFM2)	12 [217]	1-amino-3-(4-methylphenyl)-pyrido [1,2-a]benzimidazole-2,4-dicarbonitrile (iFSP1) [217]	≈1 variety of human cancer cell lines co-treatment with RSL3 [217]	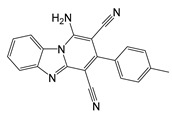
TrxR	22 [218]	5-methoxy-1-methyl-3-[(2,4,6-trifluorophenoxy)methyl]indole-4,7-dione [219]	0.034 (MIA PaCa-2) Human pancreatic cancer [219]	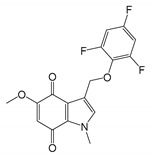
NQO1	0.79 [217]	1-isobutyl-4,6-dimethylpyrido[3,2-g]quinoline-2,5,8,10(1*H*,9*H*)-tetraone (IB-DNQ) [220]	0.08 (A549) Human Lung Cancer [220]	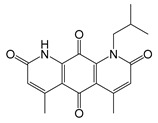
C*b*_5_R	625 [221]	N.D.	N.D.	
NADH:ubiquinone reductase Complex I	10 [222]	Rotenone [223]	0.5 > (MBA-MD-231) Triple negative breast cancer [223]	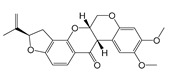
Succinate-quinone oxidoreductase Complex II	0.3 [224]	7-chloro-3-methyl-4*H*-1,2,4-benzothiadiazine 1,1-dioxide (Idra-21) [225]	0.87 [226]	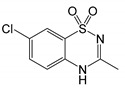
DLD	5 [227]	Huzhangoside A [228]	1.5 (A549) Human Lung Cancer [228]	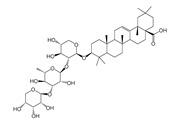
DHODH	14 [229]	2-(4-(2,6-difluorophenoxy)-3-isopropoxy-5-methyl-1*H*-pyrazol-1-yl)-5-ethylpyrimidine (BDBM50070908) [230]	0.02 (Jurkat cells) Human lymphocytes [231]	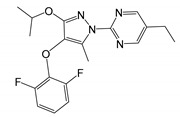

Kinetic parameters were obtained from BRENDA enzyme database [89]. N.D. Non-determined.
